# Tissue Regeneration Capacity of Extracellular Vesicles Isolated From Bone Marrow-Derived and Adipose-Derived Mesenchymal Stromal/Stem Cells

**DOI:** 10.3389/fcell.2021.648098

**Published:** 2021-02-26

**Authors:** Yuan Liu, Christina Holmes

**Affiliations:** Department of Chemical and Biomedical Engineering, Florida A&M University-Florida State University College of Engineering, Tallhassee, FL, United States

**Keywords:** mesenchymal stem cell, extracellular vesicle, bone marrow, adipose, angiogenesis, osteogenesis, immunomodulation, tissue regeneration

## Abstract

Mesenchymal stem cell (MSC)-based therapies have demonstrated tissue repair and regeneration capacity in various preclinical models. These therapeutic effects have recently been largely attributed to the paracrine effects of the MSC secretome, including proteins and extracellular vesicles (EVs). EVs are cell-secreted nano-sized vesicles with lipid bilayer membranes that facilitate cell–cell signaling. Treatments based on MSC-derived EVs are beginning to be explored as an alternative to MSC transplantation-based therapies. However, it remains to be determined which MSC source produces EVs with the greatest therapeutic potential. This review compares the tissue regeneration capacity of EVs isolated from the two most common clinical sources of adult MSCs, bone marrow and adipose tissue, with a particular focus on their angiogenic, osteogenic, and immunomodulatory potentials. Other important issues in the development of MSC-derived EV based therapies are also discussed.

## Introduction

Mesenchymal stem cell (MSC) transplantation has demonstrated great promise as a novel treatment for tissue repair and regeneration in several organ systems, including the central nervous system (CNS) (Azari et al., 2010), heart (Jeong et al., 2018), cartilage, skin, and bone (Mitxitorena et al., 2019). Displaying trophic and immunomodulatory effects upon transplantation, MSCs currently represent a critical part of clinical cell-based regenerative medicine. To date, over 950 clinical trials involving MSCs have been listed with the United States Food and Drug Administration and more than 10,000 patients have received MSC-based therapies (Pittenger et al., 2019). However, issues with MSC-based therapies, such as low cell survival rate upon transplantation, limited donor supply, donor-to-donor variability and storage issues, have prompted researchers to investigate alternative approaches. In recent years, extracellular vesicles (EVs) derived from MSCs have become the focus of much research as they exhibit many similar trophic and immunomodulatory functions. In order to translate EV-based therapies to the clinic, the relationship between MSC cell source and EV therapeutic potential needs to be clarified.

### MSCs in Tissue Repair and Regeneration

Mesenchymal stem cells are a heterogeneous subset of pluripotent stromal stem cells that are easily isolated from various tissues, including adipose tissue, peripheral blood, bone marrow, synovial fluid, muscle, placenta, umbilical cord, and dental pulp (Uccelli et al., 2008). The minimal criteria for defining MSCs are: the ability to self-renew and differentiate into classical mesodermal lineage cells such as osteoblasts, adipocytes, and chondrocytes *in vitro* and *in vivo*; a CD105+, CD73+, CD90+, and CD45–, CD34–, CD11b–, CD79a–, CD19–, and HLA class II- expression profile; a fibroblast-like morphology; and, adherence to tissue culture plastic *in vitro* (Horwitz et al., 2005; Dominici et al., 2006). Among the various MSC sources, bone marrow (BMMSCs) and adipose (ADMSCs) are the two most commonly used in preclinical and clinical tissue regeneration applications. While, umbilical cord- derived MSCs (UCMSCs) have also been widely employed in research and clinical trials, their use in many applications is limited since they are not practical for autologous administration in adults (Kern et al., 2006). Although BMMSCs were the first MSC type to be characterized and are the most widely used (Caplan, 1991), ADMSCs are an attractive alternative as they are higher in frequency, more easily obtained and cause less donor site morbidity (Reumann et al., 2018). Furthermore, ADMSCs display a higher proliferation rate than BMMSCs *in vitro* and show a greater ability to maintain their stem cell characteristics, including self-renewal, proliferation, and differentiation potential, after repeated passaging (Zhu et al., 2008).

While both BMMSCs and ADMSCs have been successfully employed in preclinical tissue repair and disease models to promote angiogenesis (Jin and Lee, 2018; Zhang et al., 2019; Ryu et al., 2020), induce bone regeneration (Jin and Lee, 2018) and modulate the immune system (Tao et al., 2016; Zhao et al., 2016; Waldner et al., 2018), there appear to be several differences between cell types. *In vitro* studies have shown that BMMSCs exhibit significantly higher chondrogenic differentiation capacity (Noël et al., 2008; Mohamed-Ahmed et al., 2018), while ADMSCs show significantly higher adipogenic capacity *in vitro* (Mohamed-Ahmed et al., 2018). ADMSCs also display a higher endothelial differentiation capacity *in vitro* than BMMSCs (Fan et al., 2016), and superior angiogenic capacity in several preclinical ischemic injury models (Ikegame et al., 2011; El-Badawy et al., 2016). However, it remains unclear which MSC source exhibits greater osteogenic capacity or immunomodulatory potential. While some *in vitro* studies showed higher osteogenic differentiation in BMMSCS than ADMSCs (Park et al., 2012), others showed the opposite (Kang et al., 2012). More significantly, no significant differences in bone regeneration ability were observed *in vivo* between the two MSC types in rat cranial defect models (Wen et al., 2013) or canine radius defect models (Kang et al., 2012). Similarly, both MSC types showed comparable immunomodulatory potential in an immunocompetent myocardial infarction (MI) model (Paul et al., 2013), while BMMSCs displayed greater immunomodulatory potential in an endotoxic shock model (Elman et al., 2014), and ADMSCs demonstrated more effective immunosuppression of peripheral blood mononuclear cells and T-cells *in vitro* (Waldner et al., 2018).

### EVs in Paracrine Signaling

While the therapeutic effects of transplanted MSCs were originally thought to be due to direct cell replacement (Friedenstein et al., 1968), research soon showed that intravenously administrated MSCs were largely caught in capillaries and/or cleared (Fischer et al., 2009), and that remaining MSCs contributed to short-term therapeutic effects (Caplan and Dennis, 2006). It is now widely theorized that the therapeutic effects of MSCs are mainly due to paracrine secretion of various growth factors, glycosaminoglycans, cytokines and EVs which modulate angiogenesis (Pankajakshan and Agrawal, 2014), apoptosis (Pan et al., 2012), proliferation (Di Nicola et al., 2002), differentiation (Chiossone et al., 2016), and the immune response (Dyer et al., 2014) to create a reparative microenvironment (Phinney and Pittenger, 2017). Secreted by the majority of cell types, EVs are phospholipid vesicles of different sizes, including micro-vesicles (MVs) (200 nm–1 μm) and exosomes (50–200 nm), that transport proteins, lipids, and nucleic acids (Hunter et al., 2008). Exosomes are generated in multivesicular bodies by the endosomal compartment and express endosomal markers (CD9, CD61, CD83, ALIX, TSG101) (Cosenza et al., 2017) and surface molecules that allow them to be targeted to recipient cells (Mathivanan et al., 2010). Meanwhile, MVs are the outcome of direct outward budding of the cell plasma membrane and thus carry cytoplasmic contents (Heijnen et al., 1999). EVs are recognized and internalized by recipient cells through receptor-ligand interactions (Raposo et al., 1996), endocytosis and/or phagocytosis (Morelli et al., 2004), or they can fuse with the target cell membrane and deliver their contents into the cytosol (Tkach and Théry, 2016). Recent research suggests that the paracrine efficacy of MSC-based therapies can largely be attributed to EVs. For example, conditioned MSC culture media was found to have therapeutic effects similar to direct delivery of MSCs in rodent models (Gnecchi et al., 2005; Aslam et al., 2009). Subsequently, Timmers et al. (2008) demonstrated that it was the EVs within the conditioned media that actually were effective.

Extracellular vesicles can be harvested via a variety of methods from cell culture media or clinical samples such as blood plasma, urine, and saliva. The most frequently employed isolation methods include differential ultracentrifugation and density gradient ultracentrifugation, both of which involve centrifugal forces greater than 100,000 × *g* and can fractionate EVs from their liquid sample of origin into subsets based on size, density, and mass (Zarovni et al., 2015; Li et al., 2017). EVs harvested from different tissues display varying content profiles, depending on their origin, age, state and environment (Tkach and Théry, 2016). For example, the microRNA (miRNA) profile of MSC-derived EVs from myoplastic syndrome patients is significantly different compared to that of EVs from disease-free patients (Muntión et al., 2016). Among EV contents, the function of bioactive lipids and proteins have been well-studied (Toh et al., 2018; Skotland et al., 2020). However, nucleic acid cargo, including mRNA, miRNAs, and other non-coding RNAs has become an increasingly hot topic in EV research. miRNAs, which are small (19–23 nucleotide) non-coding RNAs (Lau et al., 2001) that regulate gene expression via specific binding to messenger RNAs (mRNAs) (Lai, 2002), make up a large portion of the cargo within EVs (Valadi et al., 2007). miRNA transfer to recipient cells via EVs contributes significantly to paracrine signaling and has been found to be a main mediator of therapeutic effects in many preclinical studies. For example, miR-223 from BMMSCs-derived EVs contributed to cardioprotection in a surgically induced sepsis model (Wang X. et al., 2015).

The use of MSC-derived EVs in place of MSC transplantation in clinical treatments provides a number of potential advantages. EV therapies increase the accessibility of damaged tissues, since cultured MSCs are approximately 20 μm in diameter and thus tend to be caught and cleared by the circulation (Crop et al., 2010), whereas EVs are significantly smaller and have demonstrated transport through the pulmonary circulation and the blood-brain barrier (Batsali et al., 2020) (Bang and Kim, 2019). Unlike MSCS, which may undergo changes during *in vitro* culture that make them a clearance target of NK cells and macrophages (Eggenhofer et al., 2014), EVs are more likely to avoid immune rejection due to their low expression of membrane histocompatibility complexes (Lai et al., 2019). EVs are also more easily modified than MSCs to encapsulate desired therapeutic cargos, and are more easily stored than cells, since they are more stable when freezing and thawing (Lai et al., 2019). However, before the clinical application of MSC-derived EVs can be achieved, the optimal cell source for a given therapeutic application needs to be determined.

**FIGURE 1:**
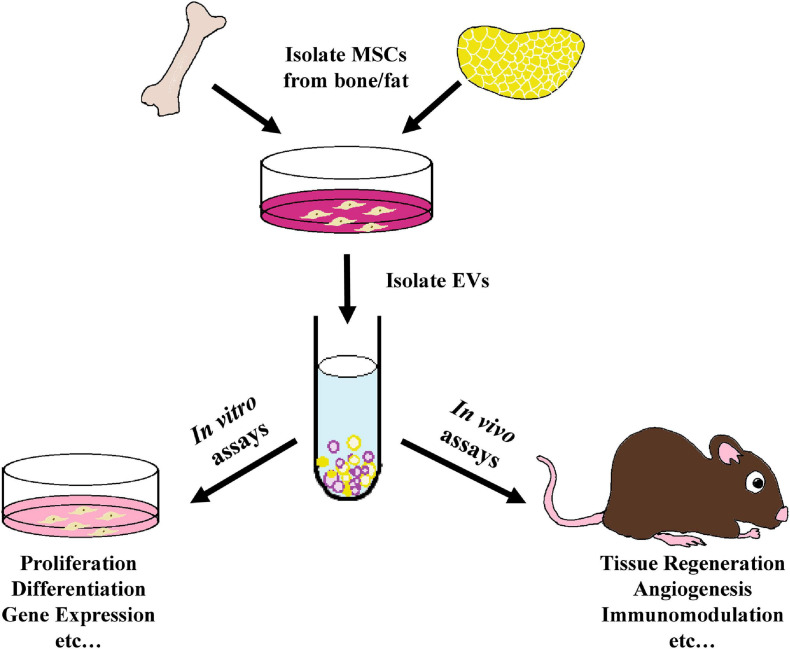
Schematic outline of the use of MSC- derived EVs within tissue regeneration models as analyzed in this review. EVs were isolated from the culture media of bone marrow-derived or adipose-derived MSCs and employed in various *in vitro* proliferation, differentiation, gene expression, and other assays, as well as within a variety of *in vivo* tissue regeneration studies, including preclinical animal models and human clinical trials.

This review will compare the therapeutic effects of EVs isolated from BMMSCs and ADMSCs in various *in vivo* tissue repair and regeneration models (Figure 1). More specifically, the capacity of BMMSC- and ADMSC-derived EVs to induce angiogenesis, osteogenesis and immunomodulation will be investigated. EV cargos and any signaling pathways involved, where characterized, will also be detailed.

## Comparing the Therapeutic Efficacy of BMMSC-Derived and ADMSC-Derived EVs

### Angiogenesis

Studies employing BMMSC-derived EVs in preclinical models, including calvarial defects (Liang et al., 2019), myocardial infarctions (MI) (Teng et al., 2015; Xu H. et al., 2020), random pattern dorsal skin flaps (Xie et al., 2019), intracerebral hemorrhages (ICH) (Han et al., 2019b), fracture non-unions (Zhang et al., 2020), focal cerebral ischemia models (Doeppner et al., 2015), traumatic brain injury (TBI) models (Zhang et al., 2017), STZ-induced diabetic rat models (Yu M. et al., 2020), and subcutaneous implantation models (Narayanan et al., 2016), generally demonstrated that EV treatment stimulated localized vasculogenesis and/or angiogenesis (see Table 1). Meanwhile, ADMSC- derived EVs promoted neovascularization and angiogenesis in fat grafting models (Han et al., 2019a), acute ischemic stroke models (Chen et al., 2016), acute kidney ischemia/reperfusion (I/R) models (Lin et al., 2016), and MI models (Xu H. et al., 2020) (see Table 1). With far fewer studies employing ADMSC-derived EVs than BMMSC-derived EVs, it remains unclear whether once source displays greater angiogenic potential than the other. In one study that directly compared the effects of EVs derived from both cell sources, human ADMSC-derived EVs displayed significantly increased therapeutic potential compared to BMMSC-derived EVs in a rat MI model, as indicated by improved cardiac function, reduced cardiomyocyte apoptosis and infarction area and increased microvessel density (Xu H. et al., 2020). By contrast, when comparing two separate brain ischemia model studies, BMMSC-derived EVs appeared to display increased angiogenic potential compared to ADMSCs-derived EVs, with the former exhibiting an approximately 4-fold increase in the number of endothelial cells compared to controls, while the latter showed a 1.5-fold change (Doeppner et al., 2015; Chen et al., 2016).

**TABLE 1:** Preclinical studies employing BMMSC- and ADMSC-derived EVs to induce angiogenesis.

**EV cell origin**	**Method of EV isolation**	**EV Characterization (size, surface markers)**	***In vitro***			***In vivo***				**Pathway(s)/ miRNA(s) involved**	**Ref.**
			**Cell and assay type**	**Amount of EVs delivered**	**In vitro effects**	**Model**	**Delivery mechanism**	**Amount of EVs delivered**	**In vivo effects**		
**Bone Marrow**											
Human BM	UC	80–182 nm CD9+, CD63+, GM130+, TSG101+	HUVECs Scratch wound, cell proliferation, and tube formation assays	50 mg/mL	Increased angiogenesis	SD rats Calvarial defect model	Implanted via porous hydroxyapatite scaffold	100 μg	Increased bone formation Increased neovascularization	PTEN; AKT/mTOR	Liang et al., 2019
Human BM p6	Centrifugation, filtration	40–100 nm CD63+, HSP70+, CD81+, CD9+	Neonatal rat cardiomyocytes cultured in hypoxic conditions ELISA	25 μg/mL	Reduced apoptosis Increased VEGF, bFGF, and HGF expression	SD rats Myocardial infarction (MI) model	Localized injection	75 μg (+1.5 × 10^6^ cells)	Increased angiogenesis Increased microvascular density	N/A	Xu H. et al., 2020
Human BM p3-p6	UC	80–100 nm CD9+, CD63+, CD81+	HBMSCs Transwell assay, qRT-PCR	5 μg/mL	Increased migration Increased VEGF, ANG1, and ANG2 expression	Wistar rats Calvarial defect model	Implanted via atelocollagen sponge	30 μg	Increased angiogenesis	N/A	Takeuchi et al., 2019
Human BM p0	N/A	N/A	HMSCs qRT-PCR, immunoblotting	EVs from 0.5 × 10^6^ cells	Increased osteogenic differentiation Increased RUNX2, Osterix, BMP9 and TGFβ1 expression (mRNA); and BMP2, TGFβ, and PDGF expression (protein)	Athymic nude mice Subcutaneous implantation	Implanted via collagen membrane	EVs from 1.25 × 10^6^ cells (+ 0.25 × 10^6^ cells)	Increased vascularization Increased expression of VEGF	N/A	Narayanan et al., 2016
Human BM >p3	PEG 6000, UC	N/A	N/A	N/A	N/A	C57BL6 mice Focal cerebral ischemia model	Femoral vein injection	EVs from 2 × 10^6^ cells	Increased angiogenesis (CD31+ cells)	N/A	Doeppner et al., 2015
Human BM p5	ExoQuick kit	CD9+, CD63+, CD81+	N/A	N/A	N/A	Wistar rats Traumatic brain injury model	Tail vein injection	100 μg (∼ 3 × 10^10^ EVs)	Increased angiogenesis (EBA/BrdU+ double labeling in endothelial cells)	N/A	Zhang et al., 2017
Human BM	Total exosome isolation reagent, centrifugation	CD63+	HUVECs Tube formation assay Cardiac myocytes TUNEL assay	N/A	Increased angiogenesis Decreased apoptosis	SD rats MI model	Localized injection	N/A	Increased angiogenesis and αSMA/CD31, cardiac function (reduced left ventricular dilation and preserved systolic function) Decreased apoptosis and infarct size	N/A	Wang L. et al., 2017
Human BM p4	UC	30-150nm CD105+, CD90+, CD73+, CD34-, CD45-	RAW264.7 cells ELISA, qRT-PCR, WB	N/A	Reduced IL-1β, TNF-α, IL-10, IL-1β, TNF-α, IL-10, Arg-1 iNOS expression Increased PTEN, AKT and p-AKT expression Melatonin (MT)-EV showed enhanced effects	SD rats STZ-induced diabetic model	SC injection	N/A	Reduced wound area size Increased angiogenesis and collagen synthesis MT-EV showed enhanced effects *also studied macrophage polarization	PTEN/AKT	Liu et al., 2020b
Human BM* p4 atorvastatin-pretreated MSCs	UC	80-120nm TSG101+, Alix+, CD81+	HUVECs Cell viability, migration, and tube formation assays	50 μg/mL	Increased cell viability, mobility, VEGF secretion Increased PDGF, EGF, and ANG1 expression	SD rats STZ-induced diabetic model	Localized injection	N/A	Accelerated wound closure Increased blood vessel area and number	miR-221-3p; AKT/eNOS	Yu M. et al., 2020
Rat BM p2-p5	UC	122nm CD90+, CD29+, CD34-, CD11b/C-	HUVECs Scratch wound, EdU incorporation, and tube formation assays	1 × 10^10^ EVs	Increased proliferation, migration, and tube formation	Wistar rats Femur fracture model	Localized injection	1 × 10^10^ EVs	Increased angiogenesis at the fracture site (3D microangiography and IHC staining)	HIF-1α-VEGF; BMP-2/Smad1/RUNX2	Zhang et al., 2020
Rat BM p3	ExoQuick-TC kit	50–100 nm CD63+	HUVECs Tube formation assay	10 μg/mL	Increased angiogenesis	SD rats Acute MI model	Localized injection	80 μg	Increased angiogenesis (new capillaries and blood vessel density)	N/A	Teng et al., 2015
Rat BM p0	ExoQuick-TC kit	N/A	N/A	N/A	N/A	Wistar rats Intracerebral hemorrhage model	Tail vein injection	100 μg	Increased vascular density and angiogenesis	N/A	Han et al., 2019b
Rat BM p4–p6	UC	TRPS: 50–150 nm, TEM: 50–100 nm CD9+, CD63+, TSG101+, GM130–	HUVECs Cell proliferation, migration, and tube formation assays	1 × 10^10^ EVs/mL	Increased proliferation, migration, and angiogenesis Normal EVs showed greater effects than diabetic EVs	SD rats Calvarial defect model	Implanted via hydrogel	1 × 10^10^ EVs/mL	Increased angiogenesis Normal EVs showed more blood vessel formation than diabetic EVs	VEGF	Zhu et al., 2019
Rat BM p4	Exosome extraction kit (E1340, Weihui Biology)	80–100 nm CD9+, CD63+, TSG101+	N/A	N/A	N/A	SD rats Random pattern dorsal skin flap model	Localized injection	135 μg	Increased angiogenesis Increased expression of VEGF and CD34	N/A	Xie et al., 2019
**Adipose**											
Human adipose* p3 cultured under normoxia and hypoxia conditions	UC	normoxia: 75 ± 61 nm hypoxia: 130 ± 65 nm CD9+, TSG101+, CD63+	HUVECs Cell proliferation, migration, transwell, and tube formation assays	25μg	Hypoxia EVs increased proliferation, migration, and tube-formation	BALB/c nude mice Fat grafting model	SC injection	50 μg	Increased neovascularization	VEGF/VEGF-R	Han et al., 2019a
Human Adipose p6	Centrifugation, filtration	30–100 nm CD63+, HSP70+, CD81+, CD9+	Neonatal rat cardiomyocytes cultured in hypoxic conditions ELISA	25 μg/mL	Reduced apoptosis Increased VEGF, bFGF, and HGF expression	SD rats MI model	Localized injection	75 μg (+1.5 × 10^6^ cells)	Increased microvascular density	N/A	Xu H. et al., 2020
Human adipose	UC	20–300 nm CD9+, CD63+, TSG101+	HUVECs Capillary formation assay	50 μg/mL	Increased capillary network formation	BALB/c nude mice Fat grafting model	SC injection	50 μg	Increased neovascularization	N/A	di Han et al., 2018
Human adipose	Total exosome isolation reagent, centrifugation	CD63+	HUVECs Tube formation assay Cardiac myocytes TUNEL assay	N/A	Increased angiogenesis Decreased apoptosis	SD rats MI model	Localized injection	N/A	Increased angiogenesis and αSMA/CD31, cardiac function (reduced left ventricular dilation and preserved systolic function) Decreased apoptosis and infarct size	N/A	Wang K. et al., 2017
Rat adipose	SDS-PAGE	CD63+, TSG101+	N/A	N/A	N/A	SD rats Acute kidney IR model	IV injection	100 μg	Increased expression of CD31, vWF, and angiopoietin	N/A	Lin et al., 2016
Pig adipose	SDS-PAGE	N/A	N/A	N/A	N/A	SD rats Acute ischemic stroke model	IV injection	100 μg	Increased protein expression of VEGF and CXCR4 Increased cellular expression of CXCR4 and SDF- 1α and endothelial function integrity (vWF)	N/A	Chen et al., 2016

Centrifugation: <100,000 *g*; HBMSCs, human bone marrow-derived mesenchymal stem cells; HMSCs, human mesenchymal stem cells; HUVECs, human umbilical vein endothelial cells; IV injection, intravenous injection, no vein specified; MI, myocardial infarction; PEG6000, polyethylene glycol 6000; Ref, references; SC injection, subcutaneous injection; SD, Sprague Dawley; STZ, streptozotocin; UC, ultracentrifugation, ≥100,000 *g*. *Cells cultured in special conditions (e.g. hypoxia).

Few of these preclinical studies have investigated the mechanisms and signaling pathways underlying the observed increase in angiogenesis induced by MSC-derived EV therapies. In a calvarial defect model, enhanced angiogenesis due to BMMSC-derived EV treatment was coupled with endogenous MSC migration (Takeuchi et al., 2019). While, in a rat full-thickness skin wound model, human BMMSC-derived EVs accelerated angiogenesis and the cutaneous wound healing process via inhibition of the TGF-β/Smad signaling pathway, as verified by RT-qPCR and western blotting analysis (Yang et al., 2018). Similarly, in a STZ-induced diabetic rat model, BMMSC-derived EVs accelerated wound closure and increased blood vessel area and number by activating the AKT/eNOS pathway (Yu M. et al., 2020). Increased angiogenesis induced by BMMSC-derived EVs is often linked to increased VEGF signaling, as was observed in a random pattern dorsal skin flap model (Xie et al., 2019) and a subcutaneous implantation model (Narayanan et al., 2016). Similarly, HIF-1α-VEGF signaling was found to be associated with enhanced angiogenesis at the fracture site in a non-union model after treatment with BMMSC-derived EVs (Zhang et al., 2020). Meanwhile, in an acute kidney I/R model (Lin et al., 2016) and an acute ischemia stroke model (Chen et al., 2016), ADMSC-derived EVs increased expression of the angiogenesis markers CD31, vWF, VEGF, CXCR4, SDF-1α, and angiopoietin. In another study that directly compared the angiogenic capacity of BMMSC-derived and ADMSC-derived EVs, similar numbers of new blood vessels were observed in a rat MI model (Wang K. et al., 2017). However, ADMSC-EVs yielded higher numbers of CD31+ cells, while EVs derived from endometrial MSCs displayed higher angiogenic capacity than EVs from either BMMSCs or ADMSCs (Wang K. et al., 2017).

*In vitro* experiments further showed that EVs isolated from both BMMSCs and ADMSCs possessed great potential for inducing angiogenesis and enabled more detailed study of the pathways underlying these effects. ADMSC-derived EVs enhanced angiogenic tube formation in human brain microvessel endothelial cells via increased expression of miR-181b-5p, which, in turn, directly targeted expression of the ion channel protein TRPM7 (Yang et al., 2018). Similarly, BMMSC-derived EVs induced angiogenic tube formation in HUVECs (Teng et al., 2015; Xie et al., 2017; Kang et al., 2020; Yu M. et al., 2020; Zhang et al., 2020) and enhanced expression of the angiogenesis-related genes VEGF, ANG1, and ANG2 in hBMMSCs (Takeuchi et al., 2019) and the mRNA expression of PDGF, EGF, and ANG1 in HUVECs (Yu M. et al., 2020). In a rare study that directly compared EVs derived from the three most commonly employed clinical MSC sources, i.e., BMMSCs, ADMSCs and UCMSCs, ADMSC-derived EVs yielded the highest *in vitro* protein expression levels of VEGF, bFGF, and HGF in rat neonatal cardiomyocytes and also showed the strongest inhibitory effect on apoptosis (Xu H. et al., 2020).

In order to improve angiogenic therapeutic capacity, many studies isolated EVs from MSCs cultured in hypoxic conditions or in the presence of dimethyloxalylglycine (DMOG), which enhances activation of HIF-1a. In a nude mouse model of fat grafting, for example, hypoxic ADMSC-derived EVs (hyp-ADSC-EVs) dramatically promoted neovascularization and increased the protein expression of VEGF/VEGF-R compared to EVs derived in normoxic conditions (Han et al., 2019a). Similarly, in other studies hyp-ADSC-EVs were found to express significantly higher levels of VEGF and VEGF-R2/R3 and promote increased HUVEC proliferation, migration and tube-formation *in vitro* (Xue et al., 2018; Han et al., 2019a), compared to EVs from cells cultured in normoxia. These hyp-ADSC-EVs also dramatically changed HUVEC expression levels of the angiogenic genes Angpt1, Flk1 and Vash1, and increased activation of the PKA signaling pathway (Xue et al., 2018). Interestingly, EVs from hypoxia-preconditioned ADMSCs exhibited increased diameters (by 59 nm) compared to those derived in normoxia (Han et al., 2019a). Meanwhile, EVs derived from DMOG-stimulated hBMMSCs increased HUVEC angiogenesis *in vitro* and decreased expression levels of PTEN (Liang et al., 2019), a tumor suppressor gene found to promote neovascularization by inducing HUVEC migration (Zhang et al., 2016). This decreased PTEN expression was further accompanied by increased expression levels of its corresponding downstream AKT/mTOR signaling pathway members, p-AKT, mTOR, and p-mTOR (Liang et al., 2019). Increased angiogenesis in a calvarial defect model treated with EVs isolated from DMOG-stimulated hBMMSCs was also observed (Liang et al., 2019).

### Osteogenesis

Although both BMMSC- and ADMSC-derived EVs have been shown to promote osteogenesis *in vivo*, most preclinical studies use BMMSC-derived EVs to induce bone formation and fracture healing. BMMSC-derived EVs were shown to promote bone regeneration in rat calvarial bone defect models (Qin et al., 2016; Takeuchi et al., 2019), murine femoral fracture models (Furuta et al., 2016; Xu T. et al., 2020), rat models of distraction osteogenesis (Jia et al., 2020), and subcutaneous bone formation models in nude mice (Narayanan et al., 2016; Xie et al., 2017) (see Table 2). By contrast, the bone regeneration capacity of ADMSCs-derived EVs has only been explored in two studies to date in calvarial defect models (Li et al., 2018); one of which involved EVs derived from hADMSCs engineered to overexpress miR-375 (Chen et al., 2019) (see Table 2). With so few studies of ADMSC-derived EVs, it is difficult to compare their osteogenic capacity to BMMSC-derived EVs. However, in separate rat calvarial defect studies, treatment with BMMSC-derived EVs led to a greater increase (fourfold) in bone volume [i.e., (BV)/(TV)] compared to controls (Qin et al., 2016), than that induced by ADMSC-derived EVs (approximately 1.33-fold) (Chen et al., 2019).

**TABLE 2:** Preclinical studies employing BMMSC- and ADMSC-derived EVs to induce osteogenesis.

**EV cell origin**	**Method of EV isolation**	**EV Characterization (Size, surface markers)**	***In vitro***			***In vivo***				**Pathway(s)/ miRNA(s) involved**	**Ref.**
			**Cell and assay type**	**Amount of EVs delivered**	***In vitro* effects**	**Model**	**Delivery mechanism**	**Amount of EVs delivered**	***In vivo* effects**		
**Bone Marrow**											
Human BM p3-p5	UC	CD63+	Human osteoblasts Osteogenic differentiation assay, qRT-PCR	5 μg/mL	Increased osteogenic differentiation Increased ALP, OCN, OPN, and RUNX2 expression	SD rats Calvarial defect model	Implanted via HyStem-HP hydrogel	100 μg	Increased bone regeneration, bone volume, and BV/TV	miR-196a	Qin et al., 2016
Human BM p3-p6	UC	80–100 nm CD9+, CD63+, CD81+	HBMSCs Transwell assay, qRT-PCR	5 μg/mL	Increased migration Increased COL I, ALP, OCN, and OPN expression	SD rats Calvarial defect model	Implanted via atelocollagen sponge	30 μg	Increased bone formation	N/A	Takeuchi et al., 2019
Human BM p4-p6	UC	80 nm CD9+, CD81+, flotillin-1-	N/A	N/A	N/A	C57BL/6 mice (WT, CD9–/–) Femoral fracture model	Localized injection	EVs from 1 × 10^5^ MSCs cultured for 2 days	Increased fracture healing, callus formation, bone union, formation of hypertrophic chondrocytes and woven bone; Increased cellular expression of TRAP and αSM	N/A	Furuta et al., 2016
Human BM p0	N/A	N/A	HMSCs qRT-PCR, immunoblotting	EVs from 0.5 × 10^6^ cells	Increased osteogenic differentiation Increased expression of RUNX2, Osterix, BMP9 and TGFβ1 (mRNA); BMP2, TGFβ, and PDGF (protein)	Athymic nude mice SC implantation model	Implanted via collagen membrane	EVs from 1.25 × 10^6^ cells (+ 0.25 × 10^6^ cells)	Increased osteogenic differentiation of naïve HMSCs Increased vascularization and expression of VEGF	N/A	Furuta et al., 2016
Rat BM p4-p6	UC	TRPS: 50–150 nm; TEM: 50–100 nm CD9+, CD63+, TSG101+, GM130-	BMSCs Cell proliferation, migration, and osteogenic differentiation assays	1 × 10^10^ EVs/mL	Increased proliferation, migration and osteogenic differentiation Normal EVs showed greater effects than diabetic EVs	SD rats Calvarial defect model	Implanted via hydrogel	1 × 10^10^ EVs/mL	Increased bone regeneration and bone volume Normal EVs showed enhanced effects compared to diabetic EVs	VEGF	Zhu et al., 2019
Rat BM p3	N/A	100-1000nm CD73+, CD105+, CD29+, CD44+, CD90+, CD34-, CD45-	HUVECs Cell proliferation, scratch wound, and tube formation assays	1, 20, or 50 μg/mL	Increased proliferation, migration, and pro-angiogenic potential	Nude mice SC implantation model	Implanted via DBM scaffold	20 μg	Increased bone regeneration, bone volume and BV/TV	N/A	Xie et al., 2017
Rat BM p3-p5	UC	TPRS: 60–130 nm; TEM: 60–100 nm CD9+, CD63+, TSG101+	BMSCs Proliferation assay, qRT-PCR	0, 1 × 10^9^, 5 × 10^9^, or 1 × 10^10^ EVs/mL	Increased proliferation, osteogenic differentiation Increased ALP, Runx2, and OCN expression	15-month SD rats Distraction osteogenesis model	Localized injection	1 × 10^10^ EVs per week	Increased new bone formation, BV/TV Improvement in mechanical tests	N/A	Jia et al., 2020
Rat BM p2-p5	UC	122 nm CD90+, CD29+, CD34, CD11b/C-	MC3T3-E1Cs EdU incorporation assay, qRT-PCR	1 × 10^10^ EVs	Increased proliferation migration, and osteogenic differentiation	Wistar rats Femoral fracture model	Localized injection	1 × 10^10^ EVs	Increased BV/TV Increased expression of BMP2, Smad1/5, RUNX2, OGN, OPN and OCN	HIF-1α-VEGF; BMP-2/Smad1/RUNX2	Zhang et al., 2020
Rat BM p3-p5	UC	50–150 nm CD81+, CD63+	MSCs Osteogenic differentiation assay, qRT-PCR	200 μg/mL	Increased osteogenic differentiation Increased Runx2, ALP and Col I expression	SD Rats Femoral fracture model	Localized injection	200 μg	Increased fracture healing, BV/TV Increased expression of Runx2, ALP and Col I	miR-128-3p/Smad5	Xu T. et al., 2020
**Adipose**											
Human adipose p1	UC	105 ± 72 nm CD63+, CD9+, tubulin-, histone1-	HBMSCs Cell proliferation, transwell, and osteogenic differentiation assays, qRT-PCR	25 μg/mL	Increased proliferation, migration, and osteogenic differentiation Increased RUNX2, ALP, and COL1A1 expression	BALB/C mice Calvarial defect model	Implanted via PLGA/pDA scaffold	165.72 ± 15.4 μg	Increased new bone formation, mature collagen formation, bone volume, recruitment of host MSCs, and expression of RUNX2 and OCN	N/A	Li et al., 2018
miRNA-375 over-expressing human ADSC	UC	75 nm CD63+, CD9+, β- tubulin-, histone1-	HBMSCs Osteogenic differentiation assay, qRT-PCR	50 μg/mL	Increased osteogenic differentiation Increased RUNX2, ALP, COL1A1 and OCN expression	SD rats Calvarial defect model	Implanted via hydrogel	1μg	Increased bone formation and BV/TV	IGFBP3	Chen et al., 2019

*BMSC, bone marrow-derived mesenchymal stem cells; BV/TV, bone volume/total volume; DBM, decalcified bone matrix; HBMSCs, human bone marrow-derived mesenchymal stem cells; HMSCs, human mesenchymal stem cells; HUVECs, human umbilical vein endothelial cells; MSC, mesenchymal stem cells; pDA, polydopamine; PLGA, poly(lactic-co-glycolic acid); SC, subcutaneous; Ref, references; SD, Sprague Dawley; UC, ultracentrifugation, ≥100,000 *g*; WT: wild type.*

The mechanisms and pathways underlying the osteogenic effects of MSC-derived EVs have not been widely reported. In a non-union model, BMMSC-derived EVs enhanced osteogenesis via the activation of the BMP-2/Smad1/RUNX2 signaling pathway (Zhang et al., 2020). In a femoral fracture model, BMMSC-derived EVs from young rats yielded increased bone formation and expression of Runx2, ALP and Col I compared to those from older rats; with EV osteogenic capacity linked inversely to levels of miR-128-3p expression, which was found to negatively modulate Smad5 signaling (Xu T. et al., 2020). Significantly, additional studies also found that miRNAs played an important role in promoting EV-mediated bone regeneration, with miR-196a regulating differentiation of osteoblasts (Qin et al., 2016), while miR-375 stimulated osteogenic differentiation of MSCs by inhibiting IGFBP3 (Chen et al., 2019).

*In vitro* studies of MSC-derived EVs further illustrated their role in bone regeneration through promotion of MSC proliferation, migration, and osteogenic differentiation (Narayanan et al., 2016; Takeuchi et al., 2019). Many studies have observed that treatment with BMMSCs-EVs increased osteogenic differentiation and upregulation of related genes, including RUNX2, Osterix, BMP9, TGF-β1, BMP2, TGF-β, OCN, ALP, Col I, and PDGF in BMMSCs (Narayanan et al., 2016; Xu T. et al., 2020) and osteoblastic MC3T3 cells (Zhang et al., 2020). Similarly, ADMSC-derived EVs were also found to promote osteogenic differentiation of human BMMSCs *in vitro* by significantly increasing the expression of RUNX2, ALP, and COL1A1 (Li et al., 2018; Chen et al., 2019). Interestingly, BMMSC-derived EVs isolated from young (2-week old) SD rats promoted proliferation and enhanced the osteogenic capacity of older BMSCs, and significantly upregulated the expression of ALP, Runx2, and OCN (Jia et al., 2020). In human osteoblasts, BMMSC-derived EVs enhanced differentiation, likely due to differential expression of miR-196a (Qin et al., 2016). In another study, miR-26 was found to be crucial to the *in vitro* capacity of BMMSC-derived EVs to induce osteogenic differentiation via silencing experiments (Luo et al., 2019).

### Immunomodulation

#### Effects on Macrophage Polarization

In preclinical models, EVs from MSCs have shown a variety of immunomodulatory effects. EVs from both BMMSCs and ADMSCs can change macrophage polarization from the pro-inflammatory M1 type to the anti-inflammatory M2 type *in vitro* and *in vivo* (see Table 3) (Li et al., 2020; Wang J. et al., 2020). With very few *in vivo* studies analyzing the effects of ADMSC-derived EVs on macrophage polarization and no direct comparison studies, it is difficult to conclude whether one EV source is more effective than the other. However, looking at separate studies that used comparable metrics, BMMSC-derived EVs induced a dramatically increased change in the expression of the M2 polarization marker CD206 (3.2-fold) in a murine acute lung injury model (Wang J. et al., 2020) compared to that induced by ADMSC-derived EVs in a murine air pouch model (1.5-fold) (Liu et al., 2020b).

**TABLE 3:** Preclinical studies employing BMMSC- and ADMSC-derived EVs to induce macrophage polarization.

**EV cell origin**	**Method of EV isolation**	**EV Characterization (Size, surface markers)**	***In vitro***			***In vivo***				**Pathway(s)/ miRNA(s) involved**	**Ref.**
			**Cell and assay type**	**Amount of EVs delivered**	**In vitro effects**	**Model**	**Delivery mechanism**	**Amount of EVs delivered**	**In vivo effects**		
**Bone Marrow**											
Human jaw BM p2-p5	ExoQuick-TC kit	20–200 nm CD63+, CD81+	Human PBMCs-derived macrophages IHC, qRT-PCR	50 μg/mL	Increased M2 polarization Increased IL-10 expression, CD14 and CD163 double-positive cells Decreased TNF-α expression	C57BL/6J mice Skin wound-healing model	IV injection	200 μg	Increased M2 polarization	miR-223	He et al., 2019
Human BM* p4 cultured w/and w/o melatonin	UC	30–150 nm CD105+, CD90+, CD73+, CD34–, CD45–	RAW264.7 cells ELISA, qRT-PCR, WB	N/A	Increased M2:M1 ratio Reduced IL-1β, TNF-α, IL-10; IL-1β, TNF-α, IL-10, Arg-1 and iNOS expression Increased PTEN, AKT and p-AKT expression Melatonin (MT)-EVs showed enhanced effects	db/db mice Air pouch model; SD rats STZ diabetic model	SC injection	N/A	Air pouch model: Increased M2:M1 ratio Diabetic model: Reduced wound area size, increased collagen synthesis MT-EVs showed enhanced effects *also studied angiogenesis	PTEN/AKT	Liu et al., 2020b
Rat BM	Total Exosome Isolation reagent; UC	CD63+, CD9+, CD81+	N/A	N/A	N/A	SD rats ICH model	Tail vein injection	100μg	Reduced neurodegeneration, neuronal apoptosis, oxidative stress, TNF-α, IL-1β, IL-6, MPO, iNOS, COX2, and MCP-1 expression miR-146a-5p overexpressing EVs showed enhanced effects	N/A	Duan et al., 2020
Rat BM p4–p8	UC; Invitrogen exosome isolation kit	30–120 nm TSG101+, CD63+, calnexin–	N/A	N/A	N/A	SD rats Spinal cord ischemia-reperfusion injury (SCIRI) model	Caudal IV injection	5 × 10^10^ EVs	Increased M2 polarization	miR-124-3p	Li et al., 2020
Mouse BM	UC	35.21 nm NALIX+, TSG101+, CD9+, CD63+	Neonatal murine ventricular myocytes (NMVMs) TUNEL assay	10 μg/μL	Reduced apoptosis	C57BL6 mice Dilated cardiomyopathy model	Tail vein injection	300 μg	Reduced IL-1, IL-6, and TNF-α expression in circulation Reduced circulating macrophages Increased M2 polarization, number of anti-inflammatory macrophages and Ly6C^*low*^ cells	AK2-STAT6	Sun et al., 2018
Mouse BM p3-p5	UC	50–150 nm TSG101+, CD9+, CD63+, CD81+	BV2 microglia and primary microglia qRT-PCR	200 μg/mL	Increased M2:M1 ratio Increased iNOS, TNF-α, IL- 1β, Arg1, CD206, and YM1/2 expression	C57BL/6 mice SCI model	Tail vein injection	200μg	Increased M2:M1 ratio	miR-216a-5p	Liu et al., 2020a
**Adipose**											
Human adipose p4–p6	UC	50–150 nm CD63+, CD81+, CD105+, CD40+, CD44+ miR-27a-3p	Bone marrow–derived macrophages (BMDM) qRT-PCR	100 μg/mL	Increased M2 polarization	C57BL/6 mice LPS-induced lung injury	Tail vein injection or intratracheal injection	50μg	Increased M2 polarization Reduced NFKB1 expression	miR-27a-3p	Wang J. et al., 2020
Mouse Adipose p4	ExoQuick-TC kit	∼100 nm TSG101+, CD9+, CD63+, HSP90+	Macrophages qRT-PCR	10 or 20 μg/mL	Increased M2 polarization Increased Arg-1 and IL-10 expression, decreased iNOS, TNF-a, and IL-12 expression	C57BL/6 mice High-fat diet model	Intraperitoneal injection	50μg	Increased WAT Beiging Reduced WAT inflammation;	N/A	Zhao et al., 2018

BV2: mouse, C57BL/6, brain, microglial cells; Centrifugation: <100,000*g*; DCM, dilated cardiomyopathy; ICH, intracerebral hemorrhage; IHC, immunohistochemistry; IV injection, intravenous injection, no vein specified; LPS, lipopolysaccharide; PBMCs, peripheral blood mononuclear cells; SC injection, subcutaneous injection; Ref, references; SCI, spinal cord injury; SD: Sprague Dawley; STZ, streptozotocin; UC, ultracentrifugation, ≥100,000 *g*; WAT, white adipose tissue; WB, Western blotting. *Cells cultured in special conditions (e.g. hypoxia).

In the preclinical models reviewed here, M2 polarization stimulated by MSC-EV treatment was found to be associated with increased expression of anti-inflammatory cytokines, such as IL-10 and TGF-β (Garnier et al., 2018), and decreased secretion of IL-6 and TNF-α (Arora et al., 2018). In a murine model of acute respiratory distress syndrome BMMSC-derived EVs reduced lung damage and LPS-induced inflammation (Deng et al., 2020). As in previous studies demonstrating that metabolic reprogramming of glycolysis in macrophages contributes to M2 polarization (Zhao et al., 2020), EV-treatment in this model was associated with downregulation of glycolysis in lung macrophages and M2 polarization (Deng et al., 2020). Research in models of cutaneous wound-healing and spinal cord ischemia-reperfusion injury (SCIRI) have also shown that BMMSC-EV induced macrophage M2 polarization was associated with the AK2-STAT6 signaling pathway (Sun et al., 2018), miR-223 (He et al., 2019), and miR-124-3p/Ern1 (Li et al., 2020). BMMSC-derived EVs also inhibited M1 microglia activation and tissue neutrophil infiltration and reduced the expression of TNF-α, IL-1β, and IL-6 in a rat ICH model (Duan et al., 2020). Similarly, ADMSC-EV treatment in a murine model of LPS-induced lung injury mitigated injury, increased localized expression of miR-27a-3p, and induced M2 macrophage polarization via NFKB1 signaling (Wang J. et al., 2020).

Interestingly, hypoxic BMMSC culture conditions were found to produce EVs with enhanced capacity to induce M2 macrophage/microglia polarization. For example, in a spinal cord injury (SCI) model, treatment with EVs from hypoxia preconditioned BMMSCs resulted in increased conversion of microglia from the pro-inflammatory M1 to the anti-inflammatory M2 phenotype, compared to EVs from BMMSCs cultured in normoxia (Liu et al., 2020a). This increased shift toward M2 polarization was found to be associated with increased expression of miR-216a-5p in hypoxic EVs. In another *in vitro* study of the microglia M1 to M2 phenotype change, hypoxic preconditioning of BMMSCs enhanced secretion of EVs and increased the M2 polarization capacity of the BMMSC secretome as compared to normoxic conditions (Yu H. et al., 2020).

#### Effects on Fibrosis

Several preclinical studies have shown that MSC-derived EVs can promote tissue regeneration over fibrosis, thus reducing the formation of scar tissue and other fibrotic processes that impair normal cellular and tissue function (Mutsaers et al., 1997) (see Table 4). For example, treatment with hBMMSC-derived EVs reduced interstitial kidney fibrosis by 80% in a mouse model of STZ-induced diabetic nephropathy, while porcine ADMSC-derived EVs resulted in a 24.4% reduction in tubulointerstitial kidney fibrosis in a porcine model of metabolic syndrome and renal artery stenosis (Eirin et al., 2017; Grange et al., 2019). BMMSC-derived EVs were also studied in two different chronic kidney injury models resulting in decreased interstitial lymphocyte infiltration in a 5/6 subtotal nephrectomy model (He et al., 2012), and significantly improved renal function and histological parameters, and reduced apoptosis and fibrotic markers in a cyclosporine nephrotoxicity model (Ramírez-Bajo et al., 2020). While, the anti-fibrotic capacity of ADMSC-derived EVs was less studied *in vivo*, *in vitro* studies showed that they inhibited the proliferation of CD4+ and CD8+ T cells (Blazquez et al., 2014), increased the expression ratios of collagen III to collagen I, TGF-β3 to TGF-β1, and MMP1 and -3 to TIMP1 in dermal fibroblasts (Wang L. et al., 2017), and prevented the transformation of tubular epithelial cells to a profibrotic phenotype via activation of tubular Sox9 (Zhu et al., 2017).

**TABLE 4:** Preclinical studies employing BMMSC- and ADMSC-derived EVs to induce anti-fibrotic effects.

**EV cell origin**	**Method of EV isolation**	**EV Characterization (Size, surface markers)**	***In vitro***			***In vivo***				**Pathway(s)/ miRNA(s) involved**	**Ref.**
			**Cell and assay type**	**Amount of EVs delivered**	***In vitro* effects**	**Model**	**Delivery mechanism**	**Amount of EVs delivered**	***In vivo* effects**		
**Bone Marrow**											
Human BM < p7	UC	162 ± 59 nm	N/A	N/A	N/A	NOD/SCID/IL2Rγ KO mice STZ-induced diabetic nephropathy model	Intravenously injection	1 × 10^10^ EVs/week, 4 weeks, 5 injections total	Reduced tubular damage, interstitial and glomerular collagen deposition Reduced collagen I, TGF-β and α-SMA (mRNA)	N/A	Grange et al., 2019
Mouse BM p2-p3	UC	100 nm	N/A	N/A	N/A	C57BL6/J mice 5/6 subtotal nephrectomy model	Caudal vein injection	30 μg	Reduced interstitial lymphocyte infiltration	N/A	He et al., 2012
**Adipose**											
Human adipose p3	ExoQuick-TC kit	CD9+, CD63+	Dermal fibroblasts qRT-PCR	100 μg/mL w/ or w/o 100 ng/mL LPS	Increased the expression ratios of collagen III to collagen I, TGF-β3 to TGF-β1, and MMP1 and −3 to TIMP1	BALB/c mice Skin wound model	IV injection	200 μg	Reduced myofibroblast differentiation, granulation tissue formation Increased ratios of collagen III to collagen I, and TGF-β3 to TGF-β1	ERK/MAPK	Wang L. et al., 2017
Human adipose p3–p6	UC	CD63+	Tubular epithelial cells (TECs) stimulated with TGF-β1 WB, qRT-PCR	N/A	Reduced transformation of renal TECs into profibrotic phenotype Reduced α-SMA, Col-I, TGF-β1, and CTGF expression	C57BL/6 mice Acute kidney injury model	Tail vein injection	100 μg	Increased tubular regeneration, and expression of Sox9 Reduced AKI and subsequent renal fibrosis	TGF-β1/Smad3	Zhu et al., 2017
Pig adipose (autologous)	UC	2/3 ∼150 nm, 1/3 ∼50 nm CD9+, CD29+, CD63+	N/A	N/A	N/A	Pigs Metabolic syndrome and renal artery stenosis model	Intrarenal injection	1 × 10^10^ EVs	Reduced ration of infiltration of M1-to-M2 macrophages Reduced MCP-1, TNF-α, IL-6, and IL-1β (protein) Increased IL-10 and IL-4 (protein)	N/A	Eirin et al., 2017

*IV injection, intravenous injection, no vein specified; Ref, references; UC, ultracentrifugation, ≥100,000 *g*.*

#### Effects on Immune Cell Infiltration

Modulation of immune cell tissue infiltration is another important target of EV-based therapies. EVs derived from both MSC sources significantly influenced immune cell migration at treatment sites in various preclinical models (see Table 5); although, again, it is difficult to make efficacy comparisons. In murine models of fat grafting (di Han et al., 2018) and high-fat diet (Zhao et al., 2018), ADMSC-derived EVs decreased inflammatory cell infiltration into adipose tissue, while, in a model of type-1 diabetes mellitus, they significantly increased the number of regulatory T cells (Tregs) (Nojehdehi et al., 2018). ADMSC-derived EVs also decreased the infiltration of mast cells, CD86+ cells, and CD206+ cells in skin lesions and reduced the mRNA expression levels of various inflammatory cytokines, such as IL-4, IL-31, and TNF-α in a murine model of atopic dermatitis (Cho et al., 2018). Similarly, BMMSC-derived EVs reduced the infiltration of CD45+ immune cells in a mouse model of aristolochic acid induced nephropathy (Kholia et al., 2020), reduced the number of GFAP+ astrocytes and CD68+ cells in a TBI model (Zhang et al., 2017) and decreased infiltration of leukocytes in a murine model of focal cerebral ischemia (Wang C. et al., 2020).

**TABLE 5:** Preclinical studies employing BMMSC- and ADMSC-derived EVs to induce immunomodulation.

**EV cell origin**	**Method of EV isolation**	**EV Characterization (Size, surface markers)**	***In vitro***			***In vivo***				**Pathway(s)/ miRNA(s) involved**	**Ref.**
			**Cell and assay type**	**Amount of EVs delivered**	**In vitro effects**	**Model**	**Delivery mechanism**	**Amount of EVs delivered**	**In vivo effects**		
**Bone Marrow**											
Human BM p2–p6	UC	35–100 nm CD63+, CD29+, CD44+, CD49e+, CD105+, CD146+, CD9+, CD81+, CD31–, CD326–, GM130–	Aristolochic Acid (AA)-treated murine tubular epithelial cells co-cultured with mouse kidney cortical fibroblasts qRT-PCR	7.5 × 10^4^ EVs/cell	Reduced α-SMA, TGFB1, and COL1A1 expression	NOD/SCID/IL2Rg KO mice Aristolochic acid nephropathy model	IV injection	1 × 10^10^ EVs/mL	Reduced infiltration of CD45+ cells	N/A	Kholia et al., 2020
Human BM p3	PEG6000, UC	ZitaView: 99–123 nm; NanoSight: 133–138 nm Syntenin+, CD63+, CD81+, CD9+, prohibition–, calnexin–	N/A	N/A	N/A	C57BL6/j mice Focal cerebral ischemia model	Tail vein injection	EV from 2 × 10^6^ MSCs	Reduced infiltration of leukocytes (PMNs, monocytes/macrophages, lymphocytes)	N/A	Wang C. et al., 2020
Human BM > p3	UC	N/A	N/A	N/A	N/A	C57BL6 mice Focal cerebral ischemia model	Femoral vein injection	EV from 2 × 10^6^ cells	Increased numbers of B-cells, natural killer cells, and T-cells in the peripheral blood	N/A	Doeppner et al., 2015
Human BM	Chromatography	N/A	N/A	N/A	N/A	C57Bl6/J mice Myocardial I/R injury model	Tail vein injection	16 μg EVs/Kg	Reduced local and systemic inflammation, local neutrophil and macrophage infiltration, and circulating WBC count	Akt/GSK3; c-JNK	Arslan et al., 2013
Human BM p5	ExoQuick kit	CD9+, CD63+, CD81+	N/A	N/A	N/A	Wistar rats Traumatic brain injury model	Tail vein injection	100 μg, ∼ 3 × 10^9^ EVs	Reduced GFAP+ astrocyte density and number of CD68+ cells	N/A	Zhang et al., 2017
Human BM < p6	UC	50–150 nm	N/A	N/A	N/A	C57Bl/6 mice Allergic Airway Inflammation model	Tail vein injection	EV from 3 × 10^6^ cells	Reduced AHR, lung inflammation, and numbers of antigen-specific CD4 T-cell (Th2 and Th17 phenotype)	N/A	Cruz et al., 2015
Rat BM p7–p9	UC	N/A	N/A	N/A	N/A	Lewis MHC disparate rats Renal allograft model	N/A	N/A	Increased T- and B-cells Reduced NK-cell infiltration and expression of TNF-α	N/A	Koch et al., 2015
Rat BM p3	ExoQuick-TC kit	50–100 nm CD63+	CD3-stimulated T-cells Proliferation assay	10 μg/mL	Decreased proliferation	SD rats Acute MI model	Localized injection	80μg	Reduced T lymphocytes	N/A	Teng et al., 2015
Rat BM	UC	30–200 nm CD9+, CD63+, CD81+	N/A	N/A	N/A	SD rats Muscle injury model	Localized injection	1 × 10^8^ EVs	Reduced expression of TGF-b	N/A	Iyer et al., 2020
Rat BM p5	QEV kit	20–130 nm CD9+, TSG101+, calnexin–	N/A	N/A	N/A	Wistar rats SCI model	Tail vein injection	100μg	Reduced complement levels and expression of NF-κB	NF-κB	Zhao et al., 2019
Rat BM p2	UC	40–100 nm CD9+, CD63+, TSG101+, calnexin–	N/A	N/A	N/A	SD rats Osteoarthritis model	Intra-articular injection	N/A	Reduced inflammation	miR-9-5p	Jin et al., 2020
Mouse BM p1–p3	UC	100–150 nm CD9+, CD63+	N/A	N/A	N/A	C57B6 mice Concanavalin A-induced liver injury model	IV injection	10 μg	Reduced expression of IL-2 (mRNA) Increased percentage of Treg to CD4+ cells among NPCs, and expression of TGFβ and HGF	N/A	Tamura et al., 2016
Mouse BM p3–p5	UC	80–150 nm CD63+, CD81+	LPS-treated MH-S alveolar macrophage cells qRT-PCR	10 μg/mL	Reduced expression of several essential glycolysis proteins: HK2, PKM2, GLUT1 and LDHA	C57BL/6 mice LPS-induced acute respiratory distress syndrome model	Intratracheal instillation	50 or 100 μg	Reduced LPS-induced inflammation, lung pathological damage, and lung tissue glycolysis	N/A	Deng et al., 2020
**Adipose**											
Human adipose	UC	20–300 nm CD9+, CD63+, TSG101+	HUVECs Capillary network formation assay	50 μg/mL	Increase angiogenic activity	BALB/c nude mice Fat grafting model	SC injection	50 μg	Reduced infiltration of inflammatory cells	N/A	di Han et al., 2018
Human adipose ≤ p9	Sequential filtration method	Most 200 nm CD9+, CD81+, TSG101+, CD63+	N/A	N/A	N/A	NC/Nga mice Atopic dermatitis model	IV or SC injection	0.14, 1.4, or 10 μg	Reduced mast cell infiltration number of CD86+ and CD206+ cells, serum IgE, and circulating eosinophils Reduced expression of IL-4, IL-23, IL-31, and TNF-α	N/A	Cho et al., 2018
Rat adipose	SDS-PAGE	N/A	N/A	N/A	N/A	SD rats Acute kidney IR model	IV injection	100 μg	Reduced expression of TNF-α, NF-κB, IL-1β, MIF, PAI-1, and Cox-2	N/A	Lin et al., 2016
Mouse adipose	UC	40–100 nm 630 mg/mL protein	N/A	N/A	N/A	C57BL/6 mice Type-1 diabetes mellitus model	Intraperitoneal injection	50 μg, twice a week	Increased number of Treg cells	N/A	Nojehdehi et al., 2018
Mouse adipose p3–p5	UC	40–100 nm CD9+, CD63+, CD81+	N/A	N/A	N/A	C57BL/6 mice Retinal laser injury model	Intravitreal injection	N/A	Reduce injury-induced inflammation and MCP-1 expression	N/A	Yu et al., 2016
Pig adipose	SDS-PAGE	N/A	N/A	N/A	N/A	SD rats Acute ischemic stroke model	IV injection	100 μg	Reduced expression of iNOS, TNF-α, NF-κB, IL-1β, MMP-9, and plasminogen activator inhibitor-1/RANTES Reduced cellular expression of CD11, CD68, glial fibrillary acid protein	N/A	Chen et al., 2016

*AHR, airway hyperreactivity; AKI, acute kidney injury; BSCB, blood-spinal cord barrier; HUVECs, human umbilical vein endothelial cells; I/R, ischemia/reperfusion; IV injection, intravenous injection, no vein specified; MHC, major histocompatibility complex; NPCs, non-parenchymal liver cells; PMNs, polymorphonuclear leukocytes; Ref, references; SC injection, subcutaneous injection; SD, Sprague Dawley; UC, ultracentrifugation, ≥100,000 *g*; WB, western blotting; WBC, white blood cells; w/, with; w/o, without.*

#### Effects on Other Immune Cells and Processes

The immunomodulatory potential of both ADMSC- and BMMSC- derived EVs has also been explored in many other preclinical disease and injury models (see Table 5). For example, ADMSC-derived EVs were found to alleviate inflammation in a retinal laser injury model (Yu et al., 2016), attenuate complement levels in a SCI model (Zhao et al., 2019), and downregulate the expression of inflammatory biomarkers in an acute ischemic stroke model (Chen et al., 2016). Meanwhile, in two kidney I/R injury models ADMSC-derived EVs either upregulated the expression of tubular Sox9 (Zhu et al., 2017), or decreased the expression of inflammatory proteins, including TNF-α, NF-κB, IL-1β, MIF, PAI-1, Cox-2 (Lin et al., 2016). Similarly, ADMSC-derived EVs mitigated scar formation, inhibited granulation tissue formation, increased expression of TGF-β3 compared to TGF-β1 and increased the ratio of collagen III to collagen I in a skin wound healing model (Wang L. et al., 2017). BMMSC-derived EVs have been studied in an even wider array of preclinical injury models than those from ADMSCs (see Table 5 and below).

Extracellular vesicles from BMMSCs have been studied in a wide array of preclinical models where immunomodulation plays a key role. Localized injection of BMMSC-derived EVs decreased inflammation in both an acute MI model (Teng et al., 2015) and an ischemia/reperfusion (I/R) model (Arslan et al., 2013) of myocardial injury. In the I/R model, both local and systemic inflammation were significantly reduced via inhibition of the c-JUK signaling pathway (Arslan et al., 2013). TGF-β expression was also shown to be reduced by BMMSC-derived EVs in a muscle injury model (Iyer et al., 2020). Similarly, treatment with BMMSC-derived EVs reduced IL2 mRNA expression, increased expression of TGF-β and HGF, and increased the ratio of Treg to CD4-positive cells among NPCs in a concanavalin A-induced liver injury model (Tamura et al., 2016). Meanwhile, in an allergic airway inflammation model induced by repeated exposure to Aspergillus hyphal extract, BMMSC-derived EVs reduced lung inflammation and airway hyperreactivity, and shifted the inflammatory response of Th2 and Th17 type T-cells (Cruz et al., 2015). In an osteoarthritis model, they reduced knee joint inflammation, mainly due to EV-expressed miR-9-5p directly targeting syndecan-1 (Jin et al., 2020). Finally, BMMSC-derived EV treatment in a renal allograft rejection model resulted in higher numbers of T- and B-cells, reduced NK-cell infiltration and significantly decreased TNFα expression (Koch et al., 2015). *In vitro* studies illustrate additional mechanism underlying the immunomodulatory effects of MSC-derived EVs. BMMSC-derived EVs significantly inhibited proliferation of CD3-stimulated T-cells (Teng et al., 2015) and increased expression of IL-10 and TGF-β1 in blood mononuclear cells, which induced Tregs differentiation and enhanced their immunosuppressive function (mo Du et al., 2018).

## Similarities and Differences in Msc-Derived EV Signaling and Therapeutic Capacity

In the reviewed studies, treatment with both ADMSC- and BMSC- derived EVs activated several common signaling pathways related to cellular survival, proliferation and/or differentiation. For example, the VEGF pathway, which is involved in angiogenesis and thus wound healing, was observed to be modulated by EVs in fracture non-union (Zhang et al., 2020), fat grafting (Han et al., 2019a), and calvarial bone defect models (Zhu et al., 2019). Several AKT-related signaling pathways, including AKT/mTOR (Liang et al., 2019), AKT/eNOS (Yu M. et al., 2020), and PTEN/AKT (Liu et al., 2020b), were also induced by ADMSC- and BMMSC- derived EVs. This is not surprising given that AKT participates in a wide range of signaling pathways, including those involved in angiogenesis, osteogenesis, and immunomodulation. Meanwhile, signaling pathways involving Smad family proteins were also activated by BMMSC- and ADMSC- derived EVs, including those involving BMP-2/Smad1/RUNX2 (Zhang et al., 2020), TGF-β1/Smad3 (Zhu et al., 2017), and Smad5 (Xu T. et al., 2020). Observed differences between studies in activated signaling pathways are due to variations in study design and purpose, including species, *in vivo* model, cell type, cell passage number, cell state, and assay type. Importantly, overlapping miRNAs within EVs or in EV-activated signaling pathways were not observed between the studies reviewed here. This may be caused by real differences in miRNA expression or variations in the methods that the researchers used to study miRNAs, such as miRNA sequencing (to target wide range of differentially expressed miRNAs) vs. qRT-PCR (to target a much smaller group of miRNAs).

While EVs from both sources showed great potential in inducing angiogenesis, osteogenesis and immunomodulation in various preclinical tissue regeneration models, the therapeutic capacity of EVs derived from ADMSC has been far less widely studied than EVs from BMMSCs. This research imbalance combined with few direct comparative studies, *in vivo* or *in vitro*, makes it difficult to conclude which EV source is best for a given application. However, *in vitro* studies do provide insight into a few possible similarities and differences in ADMSC-derived vs. BMMSC-derived EVs. For example, although EVs from both MSC sources generally expressed CD63, CD9, and CD81, and were negative for expression of either CD45, CD34, or calnexin (Katsuda et al., 2013; Del Fattore et al., 2015; Gouveia et al., 2015; Gualerzi et al., 2017; Bari et al., 2019; Chance et al., 2019; Villatoro et al., 2019; Chance et al., 2020; Hoang et al., 2020), ADMSC-derived EVs expressed higher levels of CD63, phosphatidylserine (Chance et al., 2019), and ceramides (Gualerzi et al., 2017), while BMMSC-derived EVs displayed more protein types and a higher protein content per cell (Villatoro et al., 2019). EV cargos and resulting *in vitro* effects were also observed to vary significantly depending on MSC-type. For example, ADMSC-EVS expressed higher levels of HGF, whereas BMMSC-derived EVs expressed higher levels of VEGFA, FGF-2, and PDGF-BB and thus induced greater proliferation in dermal fibroblasts (Hoang et al., 2020). In direct comparison studies, ADMSC-derived EVs were shown to promote more HUVEC tube formation (Chance et al., 2020) and display higher thrombogenic activity (Chance et al., 2019) than BMMSC-derived EVs. Meanwhile, BMMSC-derived EVs increased IL-10 secretion by a factor of 1.8 in phytohemagglutinin -activated peripheral blood mononuclear cells compared to ADMSC-derived EVs (Bari et al., 2019). Interestingly, treatment with MSC-derived EVs did not induce any effects in some *in vitro* studies, such as those on lymphocyte (Gouveia et al., 2015) and peripheral blood mononuclear cell proliferation (Villatoro et al., 2019).

## Factors and Strategies Affecting EV Therapeutic Efficacy

The method and route of EV delivery, such as intravenous injection (IV), localized injection, subcutaneous injection (SC), intraperitoneal injection (IP), intra-arterial infusion (IA), intramuscular injection (IM), topical application, or carrier-based delivery, significantly affects EV biodistribution and thus therapeutic efficacy *in vivo*. For example, in one study, IV administration lead to significantly increased BMMSC-derived EV accumulation in the liver and spleen and decreased accumulation in the gastrointestinal tract compared to SC or IP delivery; whereas IP injection lead to more EVs in the pancreas compared to IV administration (Wiklander et al., 2015). Meanwhile, IP injection of BMMSC-derived EVs was more therapeutically effective in a hepatic failure model than IV injection, resulting in a better survival rate (Haga et al., 2017). However, as systemic delivery methods, such as IV or IP injection, tend to result in EV accumulation in the liver, spleen and lungs, regardless of the cell source, delivery route, or injury model being studied (Gatti et al., 2011; Wiklander et al., 2015; Eirin et al., 2017; Maudens et al., 2018), carrier-based EV delivery methods have been developed to localize and control release (Maudens et al., 2018).

Carrier-based EV delivery methods, such as hydrogel encapsulation and surface absorption on membranes or scaffolds, provide several advantages over systemic delivery methods. Localized delivery concentrates EVs in the vicinity of target tissue, potentially reducing the amount of EVs required for achieving a given therapeutic effect, and can also prolong EV release (Liu et al., 2018; Riau et al., 2019). For example, delivery of EVs isolated from ADMSCs via a pluronic F127/oxidized hyaluronic acid-poly-ε-lysine hydrogel accelerated wound healing, promoted neovascularization, and increased collagen I and III expression in a diabetic wound healing model over a 21-day period, compared to delivery of EVs alone (Wang C. et al., 2019). Similarly, EVs loaded onto poly(lactic co-glycolic acid)/polydopamine scaffolds exhibited continuous release *in vitro*, with 28.19 ± 9.2% EVs retained within the scaffold after 8 days, and resulted in significantly improved bone regeneration when implanted *in vivo* (Li et al., 2018).

EV cargo, and thus therapeutic efficacy, is strongly influenced by a variety of factors, including donor-to-donor variability (e.g., age, gender, health status), tissue and site of cell origin (e.g., vertebral vs. femoral bone marrow), cell passage number, culture microenvironment (e.g., mechanical, chemical, hypoxia vs. normoxia), and cell state (e.g., differentiation, metabolism). For example, melatonin-treated MSCs secreted smaller sized EVs that resulted in decreased inflammation and wound size and increased angiogenesis in a diabetic wound healing model compared to EVs from untreated MSCs (Liu et al., 2020b). Meanwhile, EVs isolated from rats with type 1 diabetes yielded less bone and blood vessel formation in a rat calvarial defect model than BMMSC-EVs from normal rats (Zhu et al., 2019). Further, BMSC-EVs obtained from young donors induced increased fracture healing compared to EVs from old donors (Xu T. et al., 2020).

Many studies employ specific cell culture conditions and/or pretreatments to obtain EVs with desired cargo(s), including hypoxic conditions, drug or growth factor treatments, genetic modification, and 3D culture. These treatments have been shown to increase the therapeutic potential of the resulting EVs in several models (Haque et al., 2013; Zhang et al., 2017; Zhu et al., 2018). For example, EVs isolated from 3D cultured MSCs yielded enhanced functional recovery and immunomodulation in a traumatic brain injury model compared to EVs derived from 2D culture (Zhang et al., 2017). Similarly, EVs obtained from hypoxia preconditioned MSCs exhibited increased neovascularization and repair in a myocardial injury model compared to normoxia EVs (Zhu et al., 2018). In yet another study, IL-1β treatment increased miR-146a expression in MSCs and their corresponding EVs, which in turn resulted in increased miR-146a expression and M2 polarization in EV-treated macrophages (Song et al., 2017). However, care must be taken in using such strategies to enhance EV therapeutic efficacy as cellular pretreatment can also lead to adverse effects. For example, culturing MSCs in hypoxic conditions can interfere with differentiation and mitochondria biogenesis (Ejtehadifar et al., 2015), which, in turn can affect the therapeutic efficacy of the resulting EVs, as mitochondria were shown to be transferred via MSC-derived EVs into recipient cells (Jackson et al., 2016; Konari et al., 2019).

Post-modification of EVs is also widely used in studies to modify therapeutic efficacy. EV surfaces can be modified to facilitate uptake by specific target cells. For example, MSC-derived EVs that were surface-modified with cationic pullulan displayed increased liver targeting and resulted in improved liver function compared to unmodified EVs in a rat model of liver damage (Tamura et al., 2017). Therapeutic miRNAs and other cargos can also be introduced into EVs to improve efficacy (Naseri et al., 2018). For instance, overexpressing miR-181a in MSCs-derived EVs resulted in decreased infarct size and area-at-risk in a myocardial IR injury model compared to control EVs (Wei et al., 2019). Surface modification strategies can also be used to improve EV stability within the circulation, as glycosylation of EV surface peptides was shown to EV delivery to neuroblastoma cells (Hung and Leonard, 2015). However, post-processing of EVs can also lead to adverse effects. For example, post-processing EVs via mechanical extrusion or electroporation can result in loss of EV integrity, and biological activity (Shi et al., 2018).

## Obstacles to Clinical Translation of Msc-Derived EV Therapies

There is a crucial need for studies which directly and systematically compare EVs derived from ADMSCs and BMMSCs to determine the optimal EV source for specific clinical applications. More importantly, transferring EV-based therapies to the clinic will require the development of reproducible approaches for high-yield production of EVs with well-defined properties and therapeutic potential. Standardized EV purity metrics and isolation and characterization methods will thus be critical to enable not only systematic comparison of therapeutic EV sources, also for validation of safety and efficacy. However, standardizing characterization of even a simple parameter such as EV size can be challenging. While a wide variety of methods have been used to characterize EV size, concentration, and polydispersity, including Transmission Electron Microscopy, Atomic Force Microscopy, Nanoparticle Tracking Analysis, Tunable Resistive Pulse Sensing, and Dynamic Light Scattering (Caponnetto et al., 2017), these methods can result in different size range and concentration determinations for the same EV samples (Zhu et al., 2019; Wang C. et al., 2020), even when employing different devices based on the technology (Bachurski et al., 2019; Wang C. et al., 2020). Furthermore, different EV isolation methods can also preferentially result in different EV subpopulations, exhibiting variations in EV size distribution, yield, purity, mRNA, and protein profile (Van Deun et al., 2014; Zlotogorski-Hurvitz et al., 2015).

Extracellular vesicles storage is another important issue in expanding clinical EV treatments. For example, the combination of lyophilization and cryoprotectants was found to maintain model enzyme activity within EVs to a greater extent than lyophilization alone, or storage at 4°C and –80°C (Frank et al., 2018). Further studies to systematically characterize the dynamic changes in EV content and number for varying storage periods and conditions will be required to extend EV therapeutic use.

## Conclusion

The reviewed studies demonstrate that tissue regeneration therapies based on both BMMSC- and ADMSC-derived EVs show promise as alternatives to MSCs-based treatment. However, there is still limited evidence to determine which EV source is optimal for which tissue regeneration application, as there are significantly more studies which used BMMSC-derived EVs than ADMSC-derived EVs, few comparative studies, and considerable variation in overall study design. There is thus a crucial need for more studies, particularly *in vivo*, which directly compare the therapeutic efficacy of EVs derived from ADMSCs and BMMSCs. Optimization of donor sources, passage number, and culture conditions will also be essential to maximizing EV therapeutic capacity for specific applications. Establishment of thorough EV characterization standards, including size distribution, surface markers and cargo(s), as well as isolation and production standards will also be crucial in both systematic comparison of EV therapeutic efficacy as well as transferring EV therapies to the clinic.

## Author Contributions

YL performed the literature search and wrote the manuscript. CH revised the manuscript. Both authors reviewed the manuscript and approved the final version.

## Conflict of Interest

The authors declare that the research was conducted in the absence of any commercial or financial relationships that could be construed as a potential conflict of interest.

## Abbreviations

MSC: mesenchymal stem cells
EV: extracellular vesicles
MI: myocardial infarction
ICH: intracerebral hemorrhages
miRNA: microRNA
BMMSC: bone marrow-derived MSC
ADMSC: adipose-derived MSC
I/R: ischemia/reperfusion
SCI: spinal cord injury
Tregs: regulatory T cells.
